# Structural characterisation of the capsular polysaccharide expressed by *Burkholderia thailandensis* strain E555:: *wbiI* (pKnock-KmR) and assessment of the significance of the 2-*O*-acetyl group in immune protection

**DOI:** 10.1016/j.carres.2017.09.011

**Published:** 2017-11-27

**Authors:** Marc Bayliss, Matthew I. Donaldson, Sergey A. Nepogodiev, Giulia Pergolizzi, Andrew E. Scott, Nicholas J. Harmer, Robert A. Field, Joann L. Prior

**Affiliations:** a Chemical, Biological and Radiological Division, Defence Science and Technology Laboratory, Porton Down, Salisbury, Wiltshire, SP4 0JQ, UK; b Department of Biological Chemistry, John Innes Centre, Norwich Research Park, Norwich, NR4 7UH, UK; c Living Systems Institute, University of Exeter, Stocker Road, Exeter, EX4 4QD, UK; d University of Exeter, Stocker Road, Exeter, EX4 4QD, UK; e London School of Hygiene and Tropical Medicine, Keppler Street, London, WC1 7HT, UK

**Keywords:** Melioidosis, *Burkholderia pseudomallei*, *Burkholderia thailandensis*, Capsular polysaccharide, Glycoconjugate vaccine

## Abstract

*Burkholderia pseudomallei* and its close relative *B. mallei* are human pathogens that are classified as Tier 1 bio-threat agents. Both organisms have previously been shown to constitutively produce a capsular polysaccharide (CPS) that is both a virulence determinant and protective antigen. Extraction and purification of CPS for use as a potential vaccine candidate requires containment level 3 laboratories which is expensive and time-consuming. *B. thailandensis* strain E555 is closely related to *B. pseudomallei* and *B. mallei*, but is non-pathogenic to humans and based on immunological cross-reactivity has previously been shown to express a *B. pseudomallei*-like CPS. In this study, capsular polysaccharide isolated from an *O-*antigen deficient strain of *B. thailandensis* E555 was identified by ^1^H and ^13^C NMR spectroscopy as -3-)-2-*O*-acetyl-6-deoxy-β-d-*manno*-heptopyranose-(-1, and identical to that produced by *B. pseudomallei*. This was further substantiated by anti-CPS monoclonal antibody binding. In connection with the production of CPS fragments for use in glycoconjugate vaccines, we set out to assess the importance or otherwise of the CPS 2-OAc groups in immune protection. To this end conjugates of the native and de-*O*-acetylated CPS with the Hc fragment of tetanus toxin (TetH_c_) were used as vaccines in a mouse model of melioidosis. The level of protection provided by deacetylated CPS was significantly lower than that from native, acetylated CPS. In addition, sera from mice vaccinated with the deacetylated CPS conjugate did not recognise native CPS. This suggests that CPS extracted from *B. thailandensis* can be used as antigen and that the acetyl group is essential for protection.

## Highlights

• *B. thailandensis* E555 CPS is shown to be structurally indistinguishable from CPS expressed by *B. pseudomallei.*
• Monoclonal antibody recognition of *B. thailandensis* E555 CPS was also indistinguishable from *B. pseudomallei* CPS.
• The CPS 2-OAc group is crucial for recognition by four murine anti-CPS monoclonal antibodies.
• The 2-OAc group is also crucial for protective immunogenicity of TetH_c_-CPS in a murine model of acute melioidosis.
• *B. thailandensis* CPS is a credible and readily accessible candidate for melioidosis/glanders vaccine development.

## Graphical abstract

**Figure:**
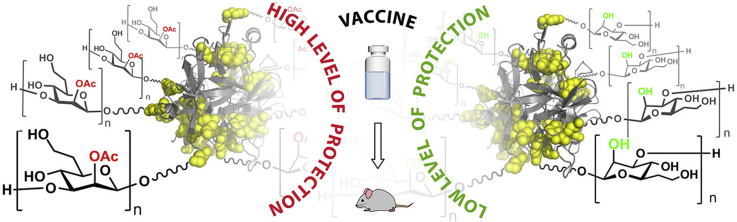

Content includes material subject to ^©^ Crown copyright (2017), Dstl. This material is licensed under the terms of the Open Government Licence except where otherwise stated. To view this licence, visit http://www.nationalarchives.gov.uk/doc/open-government-licence/version/3 or + write to the Information Policy Team, The National Archives, Kew, London TW9 4DU, or email: psi@nationalarchives.gsi.gov.uk.

## 1 Introduction

*Burkholderia pseudomallei* is a tropical pathogen and the causative agent of melioidosis, which results in an estimated 90,000 human deaths per year [1], [2]. Melioidosis can present itself in a number of forms, ranging from acute pneumonia or septicaemia to chronic infections characterised by abscess formation at multiple sites on the body. Sub-clinical infections have also been reported which may remain undiagnosed for a number of years [3], [4]. This organism is naturally resistant to a wide range of commonly used antibiotics, including penicillins, rapamycins, aminoglycosides and third-generation cephalosporins [5]. *B. mallei*, a close relative of *B. pseudomallei*, is the etiologic agent of glanders, a disease primarily of horses and related solipeds, where it is associated with a high mortality rate without antibiotic therapy [6]. Infection in humans can be very severe and is characterised by extreme pain, fever and abscesses primarily of the spleen and liver [7]. *B. pseudomallei* and *B. mallei* are classified as Tier 1 select agents by the Department of Health and Human Services [8]; no vaccines are available for either melioidosis or glanders.

Both *B. pseudomallei* and *B. mallei* organisms produce multiple cell surface glycans, one of which is common to both species; capsular polysaccharide (CPS) with the repeating homopolymer structure based on -3)-2-*O*-acetyl-6-deoxy-β-d-*manno*-heptopyranose-(1- [9]. CPS is one of the main surface-associated antigens of *B. pseudomallei* and *B. mallei* [10], with anti-CPS antibodies commonly present in convalescent patient antisera [11]. CPS is also a known virulence determinant of both of these species, as loss of capsule production results in strong attenuation in animal models of disease [3], [12]. CPS has also been demonstrated to be a protective antigen in animal models against both *B. pseudomallei* and *B. mallei* challenge; passive transfer of anti-CPS monoclonal antibody also provides partial protection [13], [14], [15]. However, despite recent advances in methods for CPS purification from pathogenic *Burkholderia,* [9] the yields are low and the requirement for high level containment facilities when cultivating these organisms at scale is problematic and costly. Several studies have, however, utilised the 6-deoxyheptan CPS extracted from non-pathogenic, select-agent excluded strains of *B. pseudomallei* [16], [17].

*Burkholderia thailandensis* is closely related to *B. pseudomallei* and *B. mallei*, but with a >10^5^-fold reduction in virulence, with recent data suggesting that *B. thailandensis* lacks a critical pathogenic activity common to *B. mallei* and *B. pseudomallei* [18], [19]. As *B. thailandensis* can be handled at lower levels of containment and is very similar genetically to *B. pseudomallei*, it is often used as a substitute in melioidosis research [4], [20], [21], [22]. Although most strains of *B. thailandensis* do not produce CPS, the E555 strain produces a polysaccharide that cross-reacts with an anti-*B. pseudomallei* CPS monoclonal antibody [23]. In combination with the high sequence similarity of six CPS genes between these two species [23], this offers the potential for a source of CPS from an essentially avirulent organism, which would be less expensive, quicker and safer to produce. However, confirmation of its CPS structure is required. Compared to non-pathogenic, select-agent excluded strains of *B. pseudomallei*; *B. thailandensis* has the advantage of being a naturally avirulent strain with no potential for reversion, which may be more palatable for use in pharmaceutical scale-up.

Whilst extraction of CPS from avirulent *B. thailandensis* E555 would be an improvement over current methods, the CPS itself is very much larger than carbohydrate antigens used in licensed vaccines which utilise oligosaccharides, and is not ideal or optimal for melioidosis glycoconjugate development considering the reported success with a synthetic hexamer of 1524 Da [24]. The use of short chain, oligosaccharide fragments of CPS would assist development of a defined glycoconjugate and provide more vaccine doses per mg of CPS extracted. While cutting-edge chemical synthesis, including of a hexasaccharide fragment of CPS has recently been reported [24], [25], such approaches remain challenging at this time. Plausible acid- or base-mediated conditions for partial depolymerisation of native CPS give rise to de-O-acetylation (unpublished observations). If the 2-OAc group could be demonstrated to have limited impact on vaccine efficacy, this would open up the use of CPS hydrolysis fragments for glycoconjugate vaccine production. A challenge to this prospect lies in recent work with synthetic CPS fragments, which show that the 2-OAc group is essential in the interaction with an anti-CPS monoclonal antibody; absence of the acetyl moiety resulted in complete loss of antibody affinity [26]. While this observation does not necessarily mean a loss of immune protection as a result of CPS deacetylation, this observation clearly merits attention. In other systems, the impact of polysaccharide antigen acetylation on immune responses is variable and has had some impact on vaccine design [27], [28], [29], [30], [31]. In the current study, we have investigated the structure of the CPS produced by *B. thailandensis* E555. Our studies have addressed the immunogenicity and the protective potential of this material conjugated to the Hc fragment of tetanus toxin (TetH_c_), a carrier protein used in previous work [24], in comparison to the corresponding deacetylated CPS conjugate in a murine model of acute melioidosis.

## 2 Results

### 2.1 6-Deoxy-d-manno-heptose

In support of analytical and structural studies on the *B. thailandensis* E555 CPS, defined 6-deoxy-*manno*-heptose was chemically synthesised. The synthetic route employed was essentially that described by Aspinall et al. [32] In outline, this involved C-6 extension of a suitably protected 6-deoxy-6-iodo-α-d-mannopyranoside with cyanide, followed by nitrile reduction-hydrolysis-reduction, proceeding via imine and aldehyde intermediates. Final global deprotection afforded the desired monosaccharide.

### 2.2 Extraction and structural characterisation of CPS from B. thailandensis E555:: wbiI (pKnock-KmR)

**Fig. 1:**
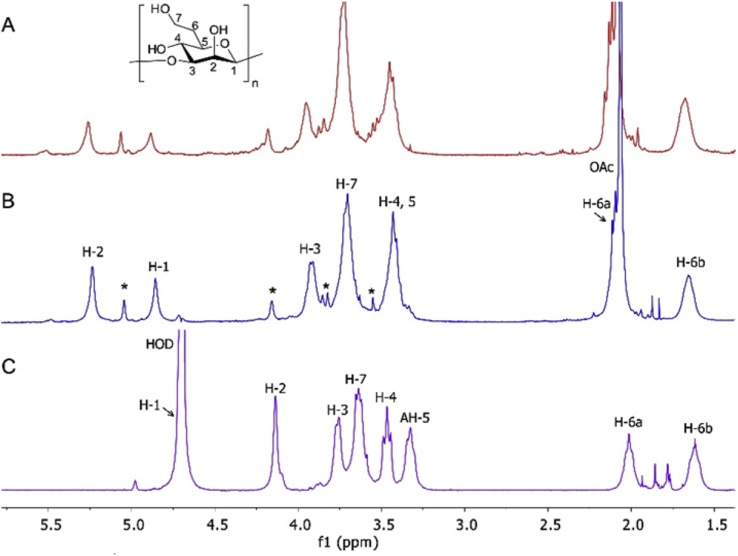
Carbohydrate regions of ^1^H NMR spectra (400 MHz, D_2_O, 25 °C) of CPS isolated from (A) *B. pseudomallei* 1026b, (B) *B. thailandensis* E555:: *wbiI* (pKnock-KmR), and (C) deacetylated CPS from *B. thailandensis* E555. Signals of α-1,3-mannan present in CPS spectra are is indicated by asterisks. Spectra of native CPS are recorded using water suppression experiment.

**Fig. 2:**
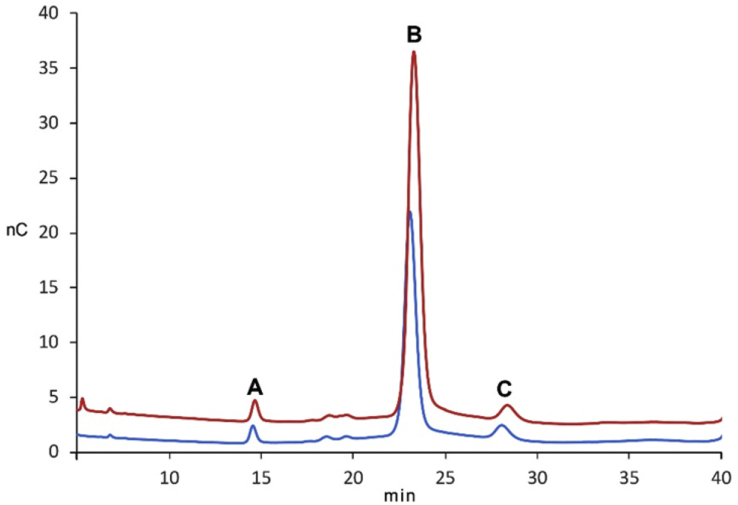
Monosaccharide analysis of *B. thailandensis* E555:: *wbiI* (pKnock-KmR) CPS by HPAE-PAD on Dionex Carbopac PA 20 column. The bottom trace shows the ion chromatogram of CPC hydrolysis products and the top one is the same sample spiked with 25 μM 6-deoxy-d-*manno*-heptose standard. Peaks correspond to (A) – unidentified sugar, (B)-6-deoxy-d-*manno*-heptose, and (C) – mannose. The analysis was performed on Dionex Carbopac PA 20 column (3 × 150 mm) at 0.25 mL/min in 8 mM NaOH at 25 °C.

Analysis of ^1^H NMR spectra of CPS extracted from *B. thailandensis* E555:: *wbiI* (pKnock-KmR) demonstrated the presence of essentially the same (1 → 3)-linked 2-*O*-acetyl-6-deoxy-β-d-*manno*-heptopyranose repeat unit as found in CPS extracted from *B. pseudomallei* strain 1026b (Fig. 1) and from *B. pseudomallei* BP2683 [9]. Corresponding HSQC and ^13^C NMR data (Figure S1 and S2 in supplementary information) also supported that structure. An additional spin system (highlighted with asterisks in Fig. 1) present in CPS ^1^H NMR spectra can be identified as a set of signals belonging to α-1,3-mannan, a polymer which is known to be produced by *B. pseudomallei* and *B. mallei* strains and is also present in purified *B. thailandensis* CPS [9]. Monosaccharide analysis of fully hydrolysed CPS from *B. thailandensis* E555:: *wbiI* (pKnock-KmR), using high-performance anion-exchange chromatography with pulse amperometric detection (HPAEC-PAD) established the presence of 6-deoxy-*manno*-heptose, which was confirmed by spiking experiment of the hydrolysed sample with defined 6-deoxy-*manno*-heptose (Fig. 2, peak B). Also identified was d-mannopyranose (peak C) by co-injection of the sample with D-mannose (data not shown).

To investigate any potential differences in CPS secondary structure, the comparative binding of an anti-CPS monoclonal antibody generated against heat-killed *B. pseudomallei* (DSTL189) to both *B. pseudomallei* and *B. thailandensis* purified CPS samples was assessed. There was no significant difference over a range of *B. pseudomallei* 1026b and *B. thailandensis* E555:: *wbiI* (pKnock-KmR) purified CPS concentrations (supplementary information, Figure S3).

### 2.3 Role of the CPS 2-OAc group in antibody recognition

After confirmation of structural similarity between *B. thailandensis* E555 and *B. pseudomallei* 1026b CPS by NMR and antibody recognition, attention turned to investigation of the influence of the acetyl moiety on CPS antibody recognition. Following recent work which demonstrated the importance of CPS acetylation to antibody recognition [26], a sample of deAc CPS was prepared by treatment of native CPS with aqueous ammonia. In these mild conditions the main structure of CPS remains unaffected as followed from monitoring the reaction by ^1^H NMR. Furthermore, the ^1^H NMR spectrum of a product of deacetylation (Fig. 1C) revealed a characteristic upfield shift of the resonance of H-2 and disappearance of OAc signal confirming a complete removal of acetyl group. Deacetylated CPS was probed with four anti-CPS monoclonal antibodies (DSTL187, 188, 189, 190)^13^ and essentially complete loss of antibody affinity to the deacetylated CPS was apparent by ELISA (supplementary information, Figure S4).

### 2.4 Role of the CPS 2-OAc group in antibody elicitation and immune protection

**Fig. 3:**
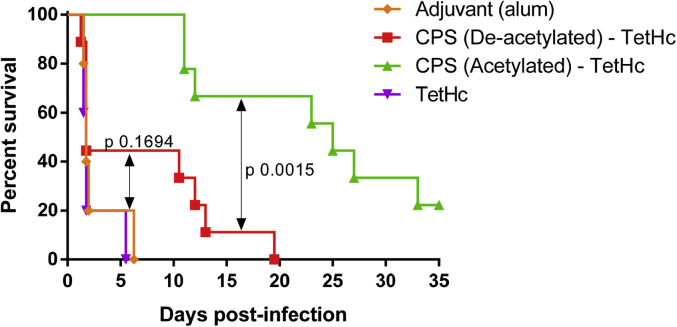
Survival of BALB/c mice vaccinated with CPS-TetH_c_ (acetylated and deacetylated CPS), TetH_c_ and alum followed by challenge with 1.17 × 10^5^ CFU via the IP route of *B. pseudomallei* K96243 (approximately 157 x median lethal doses (MLDs)). Mice were vaccinated three times in two-week intervals prior to challenge. Vaccination with acetylated CPS conjugate offered significantly greater protection than the deacetylated CPS conjugate [p = 0.0015 Log-Rank (Mantel Cox test [35])]. The deacetylated CPS-TetH_c_ conjugate did not offer any significantly greater protection than adjuvant or TetH_c_ alone [p = 0.1694 and p = 0.0872 Log-Rank, respectively (Mantel Cox test)].

Whilst CPS is a promising vaccine candidate, polysaccharide immunogenicity can be improved by conjugation to an appropriate protein carrier, such as the H_c_ fragment of tetanus toxin [33]. Such conjugates provide T-cell dependent immunogenicity, including immunological memory, avidity maturation and isotype switching to generate complement-activating antibody isotypes such as IgG1 [34]. In order to determine the effect of CPS acetylation on immune protection against *Burkholderia* infection, glycoconjugate vaccines were produced with carrier protein TetH_c_ chemically conjugated by reductive amination [16] to purified *B. thailandensis* E555:: *wbiI* (pKnock-KmR) CPS or its deacetylated counterpart (deAc CPS), which other than the absence of the *O*-acetyl group had exactly the same structure as native CPS (Fig. 1C). The glycoconjugate vaccines were analysed by SDS-PAGE and western blot (supplementary information, Figure S5-S6), and the ratio sugar: protein determined by phenol-sulfuric acid and BCA assays, respectively (supplementary information, Table S1). BALB/c mice were vaccinated with native and deAc CPS-TetH_c_ vaccines containing 10 μg of CPS per dose on three occasions, 2 weeks apart, followed by a 5-week rest period and then challenged intraperitoneally with *B. pseudomallei* K96243 (1.17 × 10^5^ CFU/mouse). There was a statistically significant difference in comparative efficacy between the TetH_c_ conjugates containing CPS or deAc CPS (p = 0.0015, Fig. 3). In addition, the protective efficacy of deAc CPS-TetH_c_ was not significantly different to adjuvant alone (p = 0.1694, Fig. 3).

**Fig. 4:**
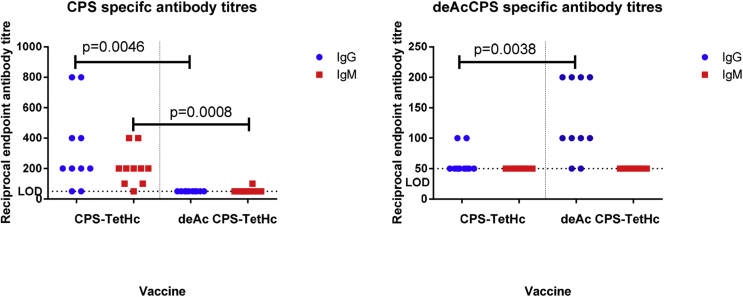
ELISA analysis of acetylated and deacetylated CPS-specific IgG and IgM immune responses following vaccination with CPS conjugate vaccines. Mice were vaccinated with native CPS-TetH_c_ or deacetylated CPS-TetH_c_ three times at 2-week intervals. Serum was obtained from mice 14 days after the third vaccination, and titres of IgG and IgM specific for CPS or deacetylated CPS were determined by ELISA. Individual symbols represent a single immunised mouse. Significance was determined by unpaired *t*-test. LOD, limit of detection.

Analysis of the relevant CPS-specific IgG and IgM titres from mouse sera following the third vaccination showed an IgG and IgM response in mice vaccinated with native CPS-TetH_c_ and only an IgG response in mice that received deAc CPS-TetH_c_. CPS-specific antibody responses in mice vaccinated with deAc CPS-TetH_c_ were significantly lower than mice that received native CPS-TetH_c_. Analysis of the deAc CPS-specific antibody response showed a very low IgG titre in native CPS-TetH_c_ vaccinated mice which was significantly lower than in mice that received deAc CPS-TetH_c_ (Fig. 4).

## 3 Discussion and conclusions

*B. pseudomallei* and its close relative *B. mallei* are Tier 1 select agents due to the severity of disease, infection by the aerosol route, antibiotic resistance and lack of available vaccines [8]. For these reasons the development of medical countermeasures against these organisms is a priority. The *manno*-heptose-based CPS expressed by *B. pseudomallei* and *B. mallei* has shown to be partially protective in animal models of melioidosis, as have anti-CPS monoclonal antibodies in passive transfer studies [13], [14]. As *B. pseudomallei* and *B. mallei* are classified by the Advisory Committee on Dangerous Pathogens (ACDP) as hazard group 3 pathogens, extraction of CPS in containment level 3 laboratories is required: this is expensive and time consuming, although several studies have described the extraction of CPS from non-pathogenic, select-agent excluded strains of *B. pseudomallei* [16], [17]. Identification of *B. thailandensis* E555 by Sim et al. [23], which expresses a *B. pseudomallei*-like CPS provides an opportunity for a less expensive, quicker route of CPS extraction and purification given the lower levels of containment required for *B. thailandensis* E555. In this study, we have shown through the use of NMR and monosaccharide analysis of hydrolysed polysaccharide that at a chemical level, the capsular polysaccharide produced from *B. thailandensis* E555 is essentially identical to that from the pathogenic *Burkholderia* species *B. pseudomallei*. Furthermore, *B. thailandensis* E555 also expresses the α-1,3 mannan found in its pathogenic relatives. Recognition of CPS extracted from *B. thailandensis* E555 with a monoclonal antibody raised against *B. pseudomallei* confirms previous work by Sim et al. [23] and infers that both polysaccharides have an epitope of similar structure. The implications of this finding mean that capsular polysaccharide can now be extracted from *B. thailandensis* E555, a naturally avirulent bacterium, which should reduce the cost of obtaining this polysaccharide and may also make licensure easier than the use of hazard group 3 bacteria, or select-agent excluded strains of *B. pseudomallei* where reversion to virulence is possible. The purity of CPS extracted from *B. thailandensis* E555 was >90% and similar to the reported purity of CPS extracted from Bp82 [16]. CPS yields from *B. thailandensis* E555 varied between 0.5 and 18 mg per litre of bacterial culture, which is a wider range than the 10–15 mg per litre of culture reported from Bp82 [16]. This may be due to slight procedural differences in the extraction method rather than a difference in CPS expression between these two species. This laboratory has not extracted CPS from *B. pseudomallei* and so a true comparison has not been made.

NMR analysis of *B. thailandensis* E555 extracted CPS shows that the sample is approximately 90% 6-deoxy-*manno*-heptose and 10% α,1-3 mannan. This composition is more in agreement with CPS produced by *B. pseudomallei* than *B. mallei*, where for the latter the ratio is approximately 43:57,^9^ indicating that *B. thailandensis* could be more suitable as a proxy for *B. pseudomallei* than *B. mallei*. This is also supported by Sim et al. [23], where PCR analysis with oligonucleotide primers against six *B. pseudomallei* CPS genes (*BPSL2791* to *BPSL2797*) were amplified in *B. thailandensis* E555. DNA sequencing of the PCR products confirmed nucleotide similarities of at least 92% to the CPS genes of *B. pseudomallei* K96243.

A solution to utilising native CPS as a melioidosis/glanders vaccine antigen may lie in producing the polysaccharide, or fragments thereof, by chemical synthesis or alternatively, naturally occurring CPS extracted from non-pathogenic *B. thailandensis* could be fragmented into minimal antigenic units (*vide infra*). This would facilitate progress of the antigen through the regulatory process, given that the vaccine candidate would be highly defined and would not originate from the pathogen. In addition, set chain lengths would lower the cost of production by increasing the number of doses per mg of CPS based on the results of a cost-benefit analysis commissioned by Dstl (unpublished). However, yields of CPS following sizing and the final vaccine carbohydrate content would have to be considered as optimal chain length and CPS content per dose is currently unknown.

In spite of the huge progresses achieved in the chemical synthesis of CPS fragments [24], [25], the CPS molecule still represents a challenge for carbohydrate chemistry, given the ambiguity surrounding whether the α-1,3-mannan and 6-deoxy-d-*manno*-heptose-based glycans are part of the same chain or are closely associated chains that are difficult to separate; in addition, the β-*manno* configuration also presents a challenge, particularly given the presence of the 2-*O*-acetyl group. As recently reported by Marchetti et al., [26] the acetyl group is required for 3C5 monoclonal antibody recognition of the molecule, although this study did not determine whether the acetyl group was important for achieving immune protection. In the current study, we found that a panel of four anti-CPS monoclonal antibodies (DSTL187, 188, 189, 190) have no binding affinity for deacetylated CPS, which supports the work by Marchetti et al. [26] and also demonstrates that these antibodies do not bind the α-1,3-mannan which is present in these samples.

Whilst the acetyl group is demonstrably important for antibody recognition, extrapolating this finding to protection against bacterial challenge is difficult on this basis alone and does not address the importance of potential antibodies raised against the CPS backbone. In the present study, we have shown that the acetyl moiety is essential for protection in an animal model of melioidosis: CPS conjugated to the carrier protein TetH_c_ provided significantly greater protection in vaccinated mice than a deacetylated CPS conjugate. Furthermore, vaccination with the deacetylated CPS conjugate offered no greater protection to bacterial challenge than vaccination with adjuvant alone. Antibody analysis of sera from mice vaccinated with the acetylated CPS conjugate showed minimal cross-reactivity to purified de-acetylated CPS, which suggests that the acetyl group is an integral part of an immunodominant epitope. The lack of an IgM antibody response to deacetylated CPS above the assay limit of detection, which was seen for CPS in this work, also suggests that the deacetylated CPS is less immunogenic. The essentiality of the CPS 2-OAc group for immune protection presents challenges for fragmenting this large (>200 kDa, as judged by PAGE) [36] polysaccharide into efficacious fragments. Both acid- or base-catalysed polysaccharide fragmentation is associated with concomitant de-O-acetylation (unpublished observations).

In summary, the use of capsular polysaccharide from *Burkholderia* species as a vaccine against melioidosis and glanders remains a tangible prospect. This study presents clear evidence that *B. thailandensis* E555 extracted CPS can be used as a substitute vaccine candidate to CPS from pathogenic species. We have shown that the acetyl group of CPS forms an immunodominant, protective, epitope which is an important finding for chemical efforts to produce synthetic CPS and also places limitations on the methods that can be employed to fragment native CPS polysaccharide.

## 4 Materials and methods

### 4.1 Bacterial growth and CPS isolation

The *O*-polysaccharide-deficient mutant of *B. thailandensis* E555 harbouring a kanamycin-resistance marked, in-frame deletion of its *wbiI* gene (*B. thailandensis* E555:: *wbiI* (pKnock-KmR)) [37] was grown in 2 L of LB broth overnight at 37 °C with shaking. Loss of *O-*polysaccharide expression had previously been confirmed by western immunoblot analysis, with bacterial stocks maintained in 35% (v/v) glycerol suspensions at −80 °C.

The CPS was extracted via a modified hot phenol method and purified as described previously [9], [38]. Briefly, the overnight culture was added in equal parts (v/v) to 90% (w/v) phenol and heated to 80 °C. The phenol was dialysed against distilled water using Spectra/Por 6–8K MWCO dialysis membrane and the resulting cell debris removed by centrifugation (10 min at 11,900×*g*). The samples were then enzymatically digested with 50 μg/mL DNAse and RNAse (Sigma) at 37 °C for 2 h and then 50 μg/mL proteinase K (Sigma) at 60 °C for 3 h. The sample was then centrifuged at 11,900×*g* for 40 min and the CPS isolated from the supernatant as precipitated gel following ultracentrifugation at 100,000×*g* for 6 h. The gel pellet was re-suspended in ultrapure water and lyophilised to concentrate. Rough LPS contaminants were then removed by acid hydrolysis with 2% (v/v) acetic acid at 100 °C for 2 h. The sample was centrifuged at 11,900×*g* for 40 min and the supernatants lyophilised to concentrate. The samples were then reconstituted at 20 mg/mL in ultrapure water and loaded onto a Sephadex G-50 column (40 cm × 2.6 cm). The purified CPS was eluted isocratically in ultrapure water at a rate of 4 mL/min. The fractions were analysed for carbohydrate using the phenol-sulphuric acid method [39] with the appropriate fractions combined and lyophilised.

CPS from *B. pseudomallei* 1026b for use as a reference in NMR analysis was kindly provided by Associate Professor P. Brett from the University of Nevada, Reno School of Medicine [9].

### 4.2 ^1^H and ^13^C NMR spectroscopy

The lyophilised CPS samples were reconstituted by dissolving in 700 μL D_2_O and NMR spectra were recorded with a Bruker Avance III HD 400 MHz spectrometer operating at 400 MHz (^1^H) or 100.6 MHz (^13^C). Assignments were made with the aid of homonuclear (COSY, NOESY) and heteronuclear (HSQC) two dimensional correlation spectroscopy.

### 4.3 Polysaccharide hydrolysis with strong acid

A solution of polysaccharide (20 μL of a 3 mg/mL solution in MQ-H_2_O) was added to a screw cap reaction vial containing trifluoroacetic acid (30 μL in 150 μL of MQ-H_2_O). The vial was sealed and heated to 100 °C for 4 h. The reaction was stopped and the solution was concentrated in *vacuo* using a SpeedVac^®^ evaporation centrifuge (Thermo scientific). The polysaccharide was reconstituted in MQ-H_2_O (300 μL), vortexed for 30 s and centrifuged for 5 min (14,000×*g*).

### 4.4 Monosaccharide analysis by HPAEC-PAD chromatography

Polysaccharide hydroxylates (10 μL) were injected onto a Dionex^®^ ICS-5000^®^ HPAEC-PAD system and the monosaccharide analysis was done using an 8 mM NaOH (Fisher^®^ HPLC grade) isocratic gradient (0.25 mL/min) for 40 min followed by a 6 min wash step (100 mM NaOH) and a 15 min re-equilibration step. Detection was achieved using pulsed amperometric detection (PAD). Spiking experiments for identification of 6-deoxy-*manno*-heptopyranose and d-mannopyranose were performed on a monosaccharide by monosaccharide basis. This was due to the impact of the addition of a monosaccharide on the retention times of the monosaccharide mixture. The polysaccharide hydroxylate samples were supplemented with monosaccharide standards (10/25 μM final concentration) and injected onto the column (10 μL).

### 4.5 Deacetylated CPS production

A sample of CPS (3 mg) in a glass sample vial was dissolved in H_2_O (0.4 mL), conc. aqueous NH_3_ (0.2 mL) was added and the vial was sealed and then heated at 50 °C for 2 h. The sample was subsequently freeze-dried and analysed by NMR. The disappearance of CPS OAc peak was observed within 80 min.

### 4.6 Generation of biotinylated monoclonal antibody DSTL189

DSTL189 antibody was diluted to 1 mg/mL in PBS. Using a syringe and needle, 1 mL of dimethylformamide (DMF) was introduced into a vial of biotin-*N*-Hydroxysulfosuccinimide (NHS) ester (Molecular Devices Corp.) and mixed until the label had completely dissolved. The dissolved label was removed using the syringe and introduced into a 1.5 mL Eppendorf tube and then placed in a foil wrapper to prevent UV degradation of the label. 10.8 μL of biotin-NHS label was then added to 1 mL of antibody solution and mixed thoroughly before incubating at room temperature for 2 h in the dark to achieve a molar coupling ration of 10. During this incubation, a PD-10 column (GE Healthcare) was equilibrated with 25 mL of PBS. A further 1–2 mL of PBS was added to the column and the bottom of the column capped (to prevent the upper disc from drying out over the remainder of the incubation period). Once incubation was complete, the cap was removed from the PD-10 column for the PBS to drain through. The 1 mL reaction volume was added to the columns, allowed to drain through and 1.25 mL of PBS added to each column to stop the reaction. Using graduated Eppendorfs to collect fractions, 3 mL of PBS was added to the column. The first 0.75 mL was collected in the first Eppendorf, 1.5 mL in the second Eppendorf and the remaining 0.75 mL in the third Eppendorf. This was repeated for the remaining reactions. The absorbance of the fractions was measured on a UV-VIS spectrophotometer at 280 nm and 362 nm. The labelled antibodies were stored at −70 °C until use.

### 4.7 Antibody analysis of purified CPS extracted from B. thailandensis E555:: wbiI (pKnock-KmR) and B. pseudomallei 1026b

A 96-well microtiter plate was coated overnight at 4 °C with 100 μL/well of purified monoclonal antibody (DSTL189) diluted to 5 μg/mL in PBS (Dulbecco's PBS 1 x without CaCl_2_, MgCl_2_). During all incubations, the microtiter plates were covered with plate sealers. Each well was washed three times with PBS-Tween 20 (0.05%) and blocked with 200 μL of PBS containing 2% (w/v) skimmed milk powder (BLOTTO) for 1 h at 37 °C. Purified CPS from *B. thailandensis* E555:: *wbiI* (pKnock-KmR)) and *B. pseudomallei* 1026b was diluted to an initial concentration of 10 μg/mL and serially diluted 1:3 in 100 μL aliquots on the plate. Following incubation for 1 h at 37 °C, each well was washed three times with PBS-Tween 20 (0.05%) and 100 μL/well of biotinylated DSTL189 antibody diluted in BLOTTO to 5 μg/mL was added. The plate was incubated for 1 h at 37 °C and each well then washed three times with PBS-Tween 20 (0.05%). A 1:1000 dilution of streptavidin peroxidase (Sigma) in 2% BLOTTO was then added and incubated at 37 °C for 1 h. Each well was washed six times with PBS-Tween 20 (0.05%) and bound conjugate detected with 100 μL/well of enzyme substrate (55 mg 2,2′-azino-bis(ethylbenzthiazoline-6-sulphonic acid) in 56 mL of 0.1 M citric acid, 44 mL of Na_2_HPO_4_ and 100 μL 30% (w/w) hydrogen peroxide, with incubation at room temperature for 15 min prior to optical density measurement at 414 nm.

### 4.8 Antibody analysis of purified CPS (acetylated and deacetylated) extracted from B. thailandensis E555::wbiI (pKnock-KmR)

A 96-well microtiter plate was coated overnight at 4 °C with 100 μL/well of *B. thailandensis* E555::*wbiI* (pKnock-KmR) purified CPS or *B. thailandensis* E555::*wbiI* (pKnock-KmR) purified deacetylated CPS at 10 μg/mL in PBS (Dulbecco's PBS 1 x, without CaCl_2_, MgCl_2_). During all incubations, the microtiter plates were covered with plate sealers. Each well was washed three times with PBS-Tween 20 (0.05%) and blocked with 200 μL of PBS containing 2% (w/v) skimmed milk powder (BLOTTO) for 1 h at 37 °C. Primary antibodies (DSTL187, 188, 189, 190)^13^ were diluted to 5 μg/mL in 2% BLOTTO and 100 μL was added to the plate in duplicate. The plate was then incubated for 1 h at 37 °C and each well washed three times with PBS-Tween 20 (0.05%). Goat anti-mouse IgG-HRP conjugate (Biorad) was diluted 1:2000 in 2% BLOTTO, 100 μL added to each well and the plate incubated at 37 °C for 1 h. Each well was washed six times with PBS-Tween 20 (0.05%) and bound conjugate detected with 100 μL/well of enzyme substrate (55 mg 2,2′-azino-bis(ethylbenzthiazoline-6-sulphonic acid)) in 56 mL of 0.1 M citric acid, 44 mL of Na_2_HPO_4_ and 100 μL 30% (w/w) hydrogen peroxide, with incubation at room temperature for 20 min prior to optical density measurement at 414 nm.

### 4.9 TetH_c_ production

TetH_c_ production was performed as previously reported [40]. Briefly, recombinant tetanus toxin H_c_ fragment was recovered from *E. coli* BL21 (pKS1-TetH_c_), kindly supplied by Dr N. Fairweather [41]. The recombinant His-tagged protein was eluted with up to 500 mM imidazole using HisTrap Columns (GE Healthcare) on an ATKA FPLC (GE Healthcare). Protein purity was assessed by Coomassie stained SDS PAGE gels (Expedeon) and protein concentration by BCA assay (Pierce).

### 4.10 CPS conjugation to TetH_c_

CPS and TetH_c_ conjugation was performed as detailed previously [16], [17]. Briefly, purified CPS was solubilised at 5 mg/mL in PBS (Sigma) and sodium *meta*-periodate (Alpha Aesar) was added to a final 30 mM concentration. The mixture was gently stirred at room temperature for 40 min and afterwards, excess oxidising agent was removed by dialysis (GeBAflex-tube maxi). To the dialysed polysaccharide solution, TetH_c_ protein was added to a final concentration of 5 mg/mL in PBS (Sigma). Sodium cyanoborohydride (Sigma: 1 M NaBH_3_CN in 10 mM NaOH) was then added at 10 μL per mL of conjugation mixture and then stirred at room temperature for 4 days. Following this, a further 10 μL of sodium borohydride (Sigma: 1 M NaBH_4_ in 10 mM NaOH) was added to each mL of conjugation mixture and stirring was continued at room temperature for 40 min. The conjugate reactions were then dialysed against distilled water (GeBAflex-tube maxi) and analysed by SDS-PAGE (12%, Expedeon) and western blot using anti-CPS monoclonal antibody DSTL189. The resulting products were then re-dissolved in ultrapure water and stored at −20 °C.

### 4.11 Conjugate analysis

CPS concentration was determined by the phenol-sulfuric acid assay [39] using 6-deoxy-*manno*-heptose to generate a standard curve and analysed against a known concentration of CPS. TetH_c_ concentration was determined by the BCA protein assay (Pierce).

### 4.12 Animal challenge

Groups of 10 BALB/c female mice between 6 and 8 weeks old (Charles River UK) were acclimatised for two weeks prior to experimental start and vaccinated via the intra-muscular (IM) route on Day 0 with either *B. thailandensis* E555 CPS conjugated to TetH_c_ or deacetylated *B. thailandensis* E555 CPS conjugated to TetH_c_, at a concentration of 10 μg CPS per mouse re-constituted in PBS and 18% v/v Alum (Invitrogen). Mice vaccinated with the acetylated CPS received 6 μg of TetH_c_ and the mice that received the deacetylated CPS: 3.3 μg TetH_c_. Groups of 5 control mice were given adjuvant or TetH_c_ only (6 μg per mouse per dose). Vaccine boosts were given on days 14 and 28 and the mice were challenged via the intra-peritoneal (IP) route with 0.1 mL of *B. pseudomallei* K96243 at 1.17 × 10^5^ cfu per mouse on day 63 [157 x median lethal dose (MLD)]. We previously determined the MLD in the BALB/c mouse model to be 744 CFU by the IP route [17]. The mice were observed twice daily for a period of 35 days after challenge when surviving mice were culled. All mice were tail-bled 2 weeks post-vaccination. Survival data was analysed with GraphPad Prism and by the Log-Rank (Mantel-Cox) test [35]. All animal work was carried out according to the Animal (Scientific Procedures) Act 1986 and following challenge, the mice were handled within a containment level 3 half-suit isolator.

### 4.13 Antibody analysis of animal sera

ELISAs were performed on sera collected 2 weeks after the third vaccination. 96 well plates were coated with either purified CPS or deacetylated CPS at 10 μg/mL in PBS (Dulbecco's PBS 1 x, without CaCl_2_, MgCl_2_) and incubated overnight at 4 °C. Each well was washed three times with PBS supplemented with 0.05% (v/v) Tween-20 (Sigma). The wells were then blocked with 2% (w/v) skimmed milk powder (Sigma) in PBS and incubated at 37 °C for 1 h. Following three further washes with PBS-Tween, two-fold dilutions of the mouse serum samples in PBS supplemented with 2% (w/v) skimmed milk powder were made across the plate. To act as a standard curve, one row of wells was coated with 5 μg/mL anti-fab antibody (Sigma), incubated and washed as described above. Appropriate isotype standards (Sigma) were diluted twofold across the plate. Also included into separate wells was serum from PBS and TetH_c_ vaccinated control mice as negative controls. The plate was incubated for a further 1 h at 37 °C and washed three times in PBS-Tween. A 1:2000 dilution of isotype specific goat anti-mouse horseradish peroxidase conjugate (Biorad) in PBS supplemented with 2% (w/v) milk powder was added to each well and the plate incubated at 37 °C for 1 h. Following six washes in PBS-Tween, 100 μL of tetramethylbenzidine (KPL) substrate was added to each well according to the manufacturer's instructions, and incubated at room temperature for 20 min prior to measuring the absorbance at 620 nm.
