# Supplementary material for: Structural characterisation of the capsular polysaccharide expressed by Burkholderia thailandensis strain E555:: wbiI (pKnock-KmR) and assessment of the significance of the 2-O-acetyl group in immune protection
Source: Carbohydr Res. 2017 Nov 27;452:17–24. doi: 10.1016/j.carres.2017.09.011 (PMC5697523; doi:10.1016/j.carres.2017.09.011)
Supplement: Supplementary [file mmc1.docx]

**Supplementary information**

Structural characterisation of the capsular polysaccharide expressed by *Burkholderia thailandensis* strain E555 :: *wbiI* (pKnock-KmR) and assessment of the significance of the 2-*O*-acetyl group in immune protection

Marc Bayliss,^a,%^ Matthew I. Donaldson,^b,%^ Sergey A. Nepogodiev,^b^ Giulia Pergolizzi,^b^ Andrew E. Scott,^a^ Nicholas J. Harmer, ^c^ Robert A. Field,^b,^*Joann L. Prior^a^ ^,^*


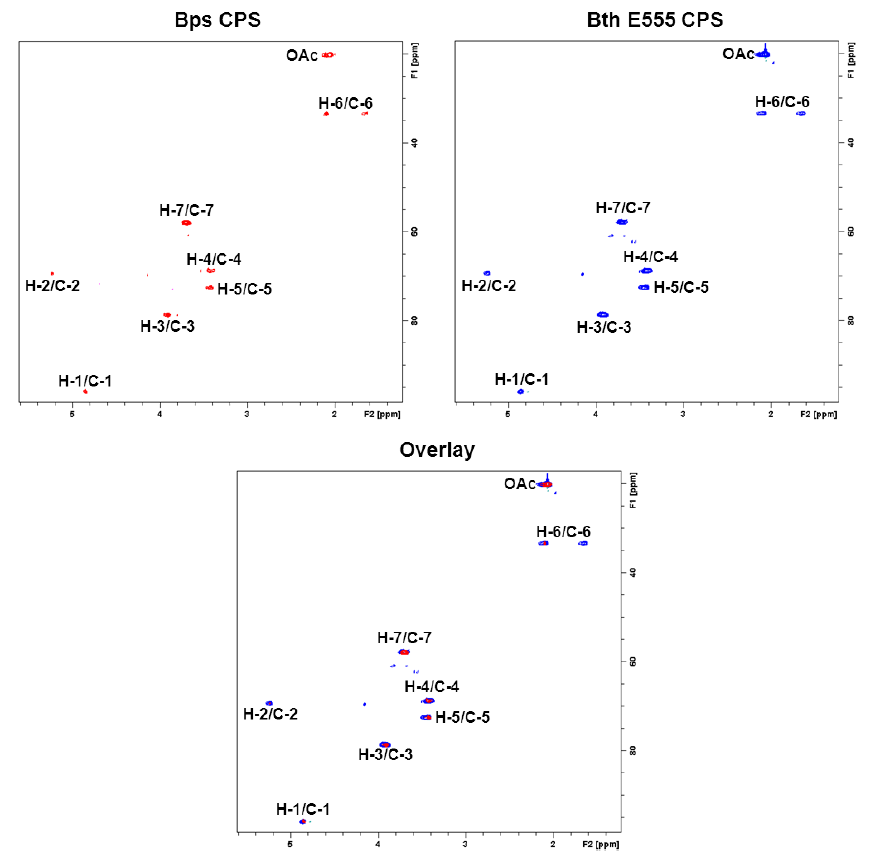


Figure S1 HSQC (D_2_O) spectra for CPS samples from *Burkholderia pseudomallei* strain 1026b (left); *B. thailandensis* strain E555 :: *wbiI* (pKnock-KmR) (right) and overlay (bottom).


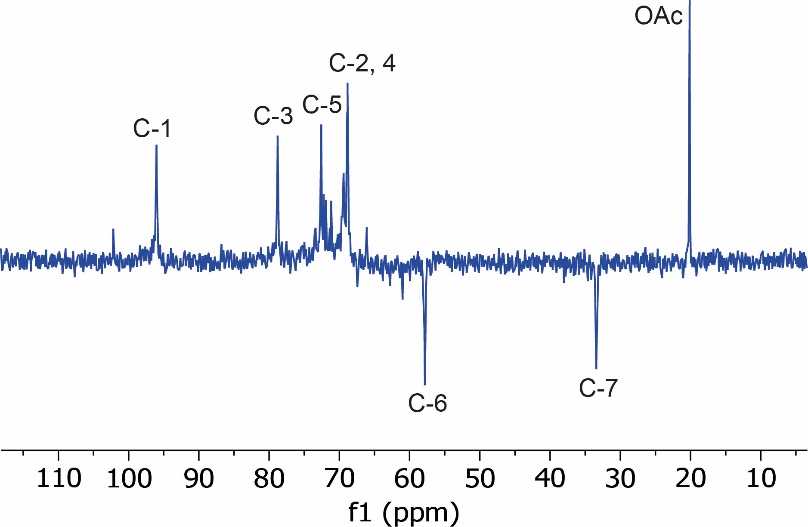


Figure S2 DEPT NMR spectrum (400 MHz, D_2_O, 25 °C) of CPS isolated from *B. thailandensis* E555 :: *wbiI* (pKnock-KmR).

Figure S3 ELISA results comparing DSTL189 CPS monoclonal antibody recognition to purified *B. pseudomallei* CPS (Bps CPS) and *B. thailandensis* E555 :: *wbiI* (pKnock-KmR) CPS (Bt CPS) over a concentration range of 10 µg/mL to 0.007 µg/mL. The data sets are not significantly different from each other (P = 0.3434 Extra sum of squares F test). Error bars = SD, n=2 at each concentration.

Figure S4 Detection of acetylated and deacetylated *B. thailandensis* E555 :: wbiI (pKnock-KmR) CPS with four anti-CPS monoclonal antibodies. Native *B. thailandensis* E555 :: wbiI (pKnock-KmR) CPS (at 10 µg/mL) and deacetylated CPS (at 10, 5, 2.5 and 1.25 µg/mL) was bound to an ELISA plate and probed with four anti-CPS monoclonal antibodies (DSTL187,188,189,and 190). (****, p ≤0.0001, 2 way ANOVA). Each value represents the mean + SD of two independent experiments.


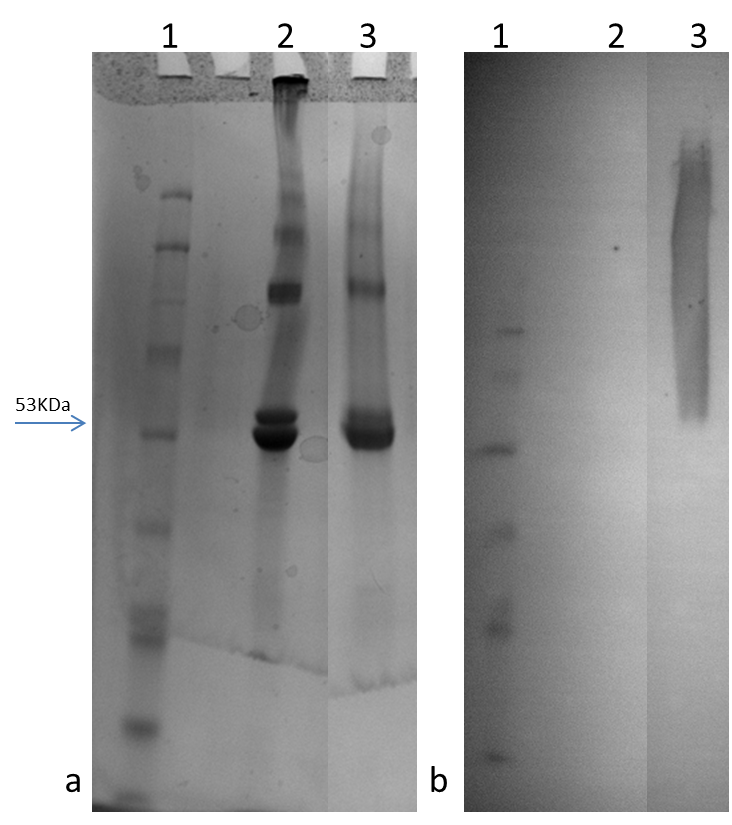


Figure S5 Native CPS-TetH_c_ conjugate. a) SDS-PAGE Coomassie staining, b) blot anti-CPS. Lane 1: ladder; lane 2: TetH_c_ (53 KDa); lane 3: CPS-TetH_c_ conjugate.


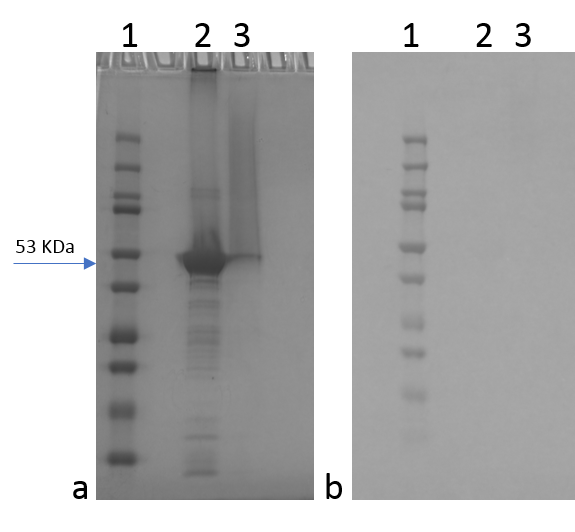


Figure S6 Deacetylated CPS-TetH_c_ conjugate. a) SDS-PAGE Coomassie staining, b) blot anti-CPS. Lane 1: ladder; lane 2: TetH_c_ (53 KDa); lane 3: deAc CPS-TetH_c_ conjugate.

|  | CPS content [μg/mL] | Protein content [μg/mL] | CPS: protein ratio |
| --- | --- | --- | --- |
| Native CPS-TetH_c_ | 404.6 | 488.6 | 0.8 |
| DeAc CPS-TetH_c_ | 750.8 | 402.9 | 1.9 |

**Table S1 CPS and protein content for native and deAc CPS-TetH_c_ conjugates.**
